# Complete Genome Sequence of *Achromobacter xylosoxidans* MN001, a Cystic Fibrosis Airway Isolate

**DOI:** 10.1128/genomeA.00947-15

**Published:** 2015-08-20

**Authors:** Jonathan P. Badalamenti, Ryan C. Hunter

**Affiliations:** a BioTechnology Institute, University of Minnesota, St. Paul, Minnesota, USA; b Department of Microbiology and Immunology, University of Minnesota, Minneapolis, Minnesota, USA

## Abstract

The genome of *Achromobacter xylosoxidans* MN001, a strain isolated from sputum derived from an adult cystic fibrosis patient, was sequenced using combined single-molecule real-time and Illumina sequencing. Assembly of the complete genome resulted in a 5,876,039-bp chromosome, representing the smallest *A. xylosoxidans* genome sequenced to date.

## GENOME ANNOUNCEMENT

*Achromobacter xylosoxidans* is an aerobic Gram-negative bacterium that is widely distributed throughout freshwater and soil environments. This bacterium is also an opportunistic human pathogen of immunocompromised hosts and is commonly associated with a range of respiratory infections. In individuals with cystic fibrosis (CF), *A. xylosoxidans* has seen an increase in prevalence, with some treatment centers reporting positive culture rates as high as 17.6% (1). Culture-independent 16S rRNA gene studies suggest that this frequency might be even higher. Despite its association with chronic lung infections and poor pulmonary function scores (2), the impact of *A. xylosoxidans* on CF disease progression is not entirely clear. This uncertainty, in addition to the bacterium’s multidrug resistance (3), robust biofilm formation (4), and transmissibility (5), warrants further study of the molecular basis for its phenotypes. Here, we report the complete genome sequence of *A. xylosoxidans* MN001—a multidrug-resistant isolate recovered from multiple patients at the University of Minnesota Cystic Fibrosis Center (institutional review board approval 1401M47262).

Genomic DNA (gDNA) was isolated from MN001 using the Wizard purification kit (Promega) and sequenced using single-molecule real-time (SMRT) and Illumina technologies. SMRT libraries were constructed according to Pacific Biosciences protocols with a 20-kb insert size. Following ligation of SMRTbell adapters, sheared gDNA was size selected with a 4-kb cutoff using Blue Pippin electrophoresis (Sage Science) to generate a greater fraction of long reads capable of resolving repeat sequencing in the *A. xylosoxidans* genome. Sequencing was performed using the PacBio RS II platform. Subread filtering from 2 SMRTcells captured with a 240-min movie and P6-C4 chemistry yielded 592 Mbp of sequence reads with an average read length of 11,520 bp (*N*_50_, 16,106 bp). Assembly was performed using the Hierarchical Genome Assembly Process (HGAP) version 3 (6) in SMRT Analysis version 2.2 hosted on the University of Minnesota supercomputer. Remaining indels were removed with three successive passes through Quiver to achieve a final consensus accuracy of >99.9997% (QV 56) at 100× coverage. This assembly consisted of one circular contig representing a 5,876,039-bp chromosome. Illumina libraries were analyzed using MiSeq with 250-bp paired-end sequences, yielding ~2.5 million reads. Reads were mapped onto the SMRT-derived contig using breseq version 0.24rc6 (7), and the 10 indels remaining after polishing were corrected using Pilon version 1.10 (8), yielding essentially perfect final per-base accuracy. The MN001 genome was annotated with Prokka version 1.11 (9) using *A. xylosoxidans* NH44874-1996 (4) as the reference genome.

At 5.8 Mbp, the MN001 genome is remarkably smaller than the seven *A. xylosoxidans* genomes published to date. The G+C content is 67.72%, which is consistent with previously sequenced strains. A total of 5,328 genes, including 10 rRNA (3 16S, 3 23S, 4 5S) and 67 tRNA genes, were annotated. Function was assigned for 4,333 of 5,251 predicted coding sequences (>82%). Detailed analyses of the MN001 genome, including comparative studies with other *A. xylosoxidans* strains are in progress.

### Nucleotide sequence accession numbers.

The assembly and annotation have been deposited in GenBank under the accession number CP012046. PacBio and Illumina reads have been deposited to the NCBI Sequence Read Archive under BioProject number PRJNA288995.
